# BCHE as a Prognostic Biomarker in Endometrial Cancer and Its Correlation with Immunity

**DOI:** 10.1155/2022/6051092

**Published:** 2022-07-21

**Authors:** Junxiu Liu, Tian Tian, Xiangyu Liu, Zhumei Cui

**Affiliations:** Department of Obstetrics and Gynecology, The Affiliated Hospital of Qingdao University, Qingdao, Shandong, China

## Abstract

**Background:**

In developed countries, the most common gynecologic malignancy is endometrial carcinoma (EC), making the identification of EC biomarkers extremely essential. As a natural enzyme, butyrylcholinesterase (BCHE) is found in hepatocytes and plasma. There is a strong correlation between BCHE gene mutations and cancers and other diseases. The aim of this study was to analyze the role of BCHE in patients with EC.

**Methods:**

A variety of analyses were conducted on The Cancer Genome Atlas (TCGA) data, including differential expression analysis, enrichment analysis, immunity, clinicopathology, and survival analysis. The Gene Expression Omnibus (GEO) database was used to validate outcomes. Using R tools, Gene Set Enrichment Analysis (GSEA) and Gene Ontology (GO) analyses revealed the potential mechanisms of BCHE in EC. Sangerbox tools were used to delve into the relations between BCHE expression and tumor microenvironment, including microsatellite instability (MSI), tumor neoantigen count (TNC), and tumor mutation burden (TMB). BCHE's genetic alteration analysis was conducted by cBioPortal. In addition, the Human Protein Atlas (HPA) was used to validate the outcomes by immunohistochemistry, and an analysis of the protein-protein interaction network (PPI) was performed with the help of the STRING database.

**Results:**

Based on our results, BCHE was a significant independent prognostic factor for patients with EC. The prognosis with EC was affected by age, stage, grade, histological type, and BCHE. GSEA showed that BCHE was closely related to pathways regulating immune response, including transforming growth factor-*β* (TGF-*β*) signaling pathways and cancer immunotherapy through PD1 blockade pathways. The immune analysis revealed that CD4+ regulatory T cells (Tregs) were negatively correlated with BCHE expression and the immune checkpoint molecules CD28, ADORA2A, BTNL2, and TNFRSF18 were all significantly related to BCHE. BCHE expression was also associated with TMB by genetic alteration analysis.

**Conclusions:**

Identifying BCHE as a biomarker for EC might help predict its prognosis and could have important implications for immunotherapy.

## 1. Introduction

EC is the fourth most frequent malignancy in women, and the incidence of death from EC ranked sixth [1]. Alarmingly, the incidence of EC increased and the 5-year survival decreased in recent years [2]. Early EC patients will have a better prognosis with 5-year overall survival rate over 74%. However, in some cases, endometrial cancer can progress to an advanced stage before symptoms appear, which results in a worse prognosis [3]. Most EC-related deaths can be attributed to the lack of early diagnostic and therapeutic biomarkers [4]. Consequently, identifying specific biomarkers for EC remains challenging.

Butyrylcholinesterase (BCHE) belongs to the family of alpha-glycoproteins and presents in the nervous system and liver [5]. In many clinical conditions, such as liver damage, inflammation, infection, and malignancy, its serum level is reduced [6]. Studies have shown a correlation of BCHE with cell adhesion, cell differentiation, apoptosis, and tumorigenesis [7, 8]. BCHE may represent one such protein marker in some tumors such as breast cancer [9, 10], colorectal carcinoma [11], oral squamous cell carcinoma [12], and lung squamous cell carcinoma [13]. Nevertheless, no research has been conducted on BCHE's role in endometrial cancer.

Data obtained from TCGA were used to examine the BCHE expression in EC samples, and the HPA was used to verify its protein expression. We investigated the relationship between the expression of BCHE and prognosis and clinical parameters by using R (Version 4.0.2). To provide a greater understanding of BCHE regulatory mechanisms in EC, we performed GSEA and GO analyses. In addition to these, using the R and Sangerbox tools, we investigated the associations between BCHE and the tumor microenvironment and immune system. A nomogram was created to predict overall survival (OS) probabilities in EC; the performance and accuracy of this nomogram were evaluated using calibration curves and the receiver operating characteristic (ROC) curve. Finally, we explored the genetic alterations of BCHE in EC samples.

The association of BCHE with endometrial cancer is firstly analyzed in depth. According to our work, increased expression of BCHE was associated with poor overall survival. GSEA showed pathways enriched in our results were closely linked to immune response which contained the TGF-*β* pathway. In addition, we found a negative correlation between BCHE and Tregs. The mutations of BCHE were also significantly related to the tumor immune system. A comprehensive understanding of BCHE potential mechanisms and roles in EC is presented, which could contribute to the understanding of EC mechanisms. The work design and flowchart of this study are given in Figure S1.

## 2. Materials and Methods

### 2.1. Data Collection and Differential Expression Analysis

BCHE expression levels of TCGA pan-cancer were downloaded from the UCSC Xena database (https://xenabrowser.net/datapages/). By utilizing TCGA database for endometrial cancer, we obtained information about BCHE expression levels and relevant clinical information (Data Type: Clinical Supplement) [14]. Our research excluded duplicated samples. In addition, to validate the BCHE mRNA expression in patients with EC, the raw gene profiles of GSE17025 [15] and GSE63678 [16] were obtained from the Gene Expression Omnibus (GEO) database. The differential expression analysis was visualized by “ggplot2” R package (Version 4.0.2; https://www.r-project.org/). The HPA (http://www.proteinatlas.org) database contains information about the expression and localization of human proteins in various tissues and organs [17]. Online immunohistochemical staining data offered by HPA was used to analyze whether BCHE protein expression was different between normal and EC tissues. Scatterplots using clinicopathological parameters as variables were visualized to demonstrate the relationship between the expression of BCHE and clinicopathological parameters. Logistic regression was used by R tools to analyze the relationship between clinical characteristics and BCHE expression.

### 2.2. Survival Analysis

In order to identify independent prognostic factors for EC, we used univariate and multivariate Cox regression analyses conducted by R packages “survival” and showed it as a forest map by “ggplot2.” The risk factor graph was drawn based on the risk score calculated by Cox regression model and prognosis (survival). In order to estimate BCHE predictive power in diagnosing EC and normal, we used “pROC” to analyze data and “ggplot2” to draw ROC curve. Through the use of the “survminer” R package, it was possible to perform a K-M survival analysis to determine the relationship between BCHE expression and the survival days of EC patients. A *p* value of less than 0.05 was regarded as statistically significant. For overall survival prediction, the “rms” and “survivalROC” packages in R were used to create a nomogram based on Cox regression model, and the area under curve (AUC) value was calculated to evaluate its performance. For assessing the performance of the constructed nomogram, calibration curves were also used [18].

### 2.3. Functional Enrichment Analysis

We performed a differential expression analysis between high and low expression of BCHE in EC from TCGA using “DESeq2” R package, and genes with a *p* value < 0.05 were considered differentially expressed [19]. GO enrichment analysis consists of biological processes (BP), molecular functions (MF), and cellular components (CC). Using “clusterProfiler” R package, we performed the GO analysis of BCHE. A *p* value of less than 0.05 was regarded as statistically significant [20]. Additionally, GSEA was conducted on normalized BCHE RNA-Seq data from TCGA by using “clusterProfiler” and the number of permutations was set to 1000 [21]. Using “GSEA,” we analyzed the Kyoto Encyclopedia of Genes and Genomes (KEGG), REACTOME, Pathway Interaction Database (PID), and Wiki Pathways (WP) to explore BCHE's possible biological functions [22]. Enrichment results must satisfy two conditions to be considered statistically significant: a nominal *p* value < 0.05 and a false discovery rate (FDR) < 0.25.

### 2.4. Immune-Related Analysis

We investigated the relationship between BCHE expression and immune cells by using “GSVA” R packages [23, 24]. We established gene expression datasets with standard annotation files and applied 1000 permutations to the default signature matrix. According to the median BCHE expression level, we divided TCGA data into two groups (high and low) to determine the level of immune cell infiltration. Sangerbox (http://www.sangerbox.com/tool) is a comprehensive resource for systematic analysis of immune infiltrates across diverse cancer types. As a result of analyzing the BCHE expression matrices using the Sangerbox tools, we calculated the Immune Score, Stromal Score, and Estimate Score. Through querying BCHE gene in “immune checkpoint gene analysis” module of Sangerbox, the visualization of immune checkpoint molecules of pan-cancer was also presented.

### 2.5. Analysis of Genetic Alteration by cBioPortal

By searching the cBioPortal database (http://cbioportal.org), we explored BCHE alteration frequency, copy number alteration (CNA), structural variant, and mutation type via TCGA-UCEC pan-cancer atlas studies by querying the BCHE gene. Additionally, survival differences between BCHE genotypes were presented by K-M plots [21, 25].

### 2.6. Protein-Protein Interaction (PPI), Tumor Mutational Burden (TMB), Tumor Neoantigen Count (TNC), and Microsatellite Instability (MSI)

Using the online STRING (https://string-db.org/) database [26], an analysis of the PPI network was also carried out to find potential relationships between BCHE and other genes in EC by querying BCHE gene in Homo sapiens. In order to explore the relationship between BCHE gene expression and TMB, MSI, and TNC, correlation analyses were conducted by querying BCHE gene in single-gene pan-cancer analysis tool of Sangerbox.

## 3. Results

### 3.1. BCHE Expression Analysis

**Figure 1:**
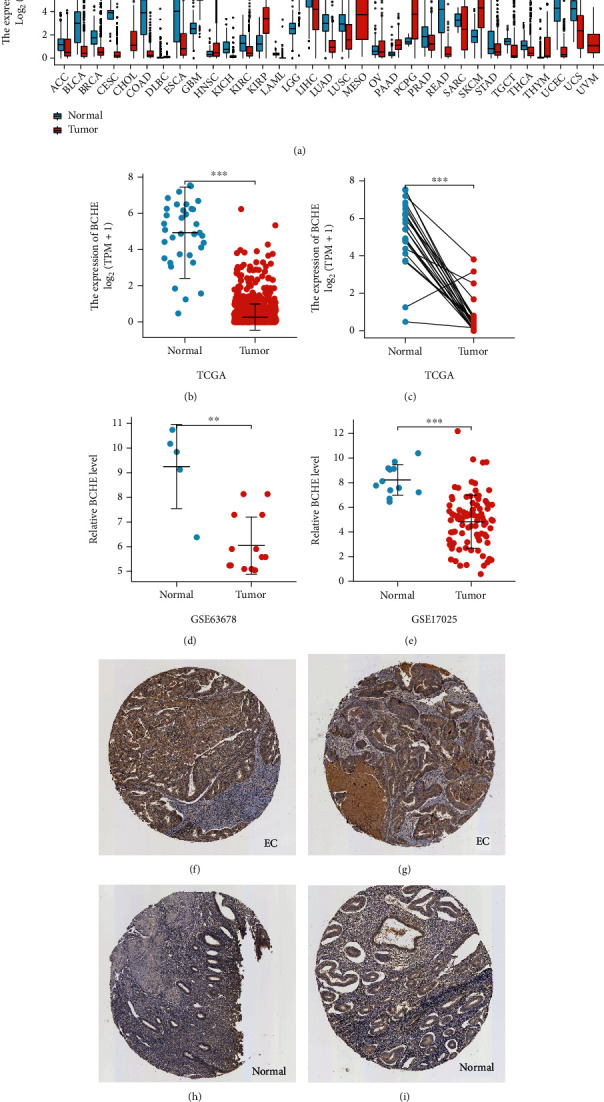
BCHE expression analysis. (a) BCHE expression in normal and tumor tissues in TCGA and GTEx pan-cancer data. (b) BCHE expression in unpaired EC samples. (c) BCHE expression in paired EC samples. (d) BCHE expression in GSE63678. (e) BCHE expression in GSE17025. (f, g) Representative images of immunohistochemistry showing BCHE expression in endometrial carcinoma tissues. (h, i) Representative images of immunohistochemistry showing BCHE expression in normal endometrial tissues.

In the first place, we examined pan-cancer data from TCGA and GTEx to assess BCHE expression. In 20 kinds of cancer, BCHE expression was lower than normal; in contrast, its expression was high in 7 kinds of cancer. The details are depicted in Figure 1(a), and TCGA tumor abbreviations are listed in Supplementary Table 1. Based on the data from TCGA-UCEC, the expressions of BCHE in 35 normal and 543 EC samples and 23 paired samples were plotted on scatter plots (Figures 1(b) and 1(c)) and our analysis revealed the decrease of BCHE expression in EC tissues (*p* < 0.001). In addition, we also downloaded the microarray data from GEO databases, GSE63678 and GSE17025, to verify the above results. The results also illustrated that BCHE levels were lower in endometrial carcinoma when compared to normal tissues (Figures 1(d) and 1(e)). We further confirmed the expression of BCHE in EC via immunohistochemistry from the HPA database (Figures 1(f)–1(i)), showing the same result.

### 3.2. Relationship between BCHE Expression and Clinicopathology

**Figure 2:**
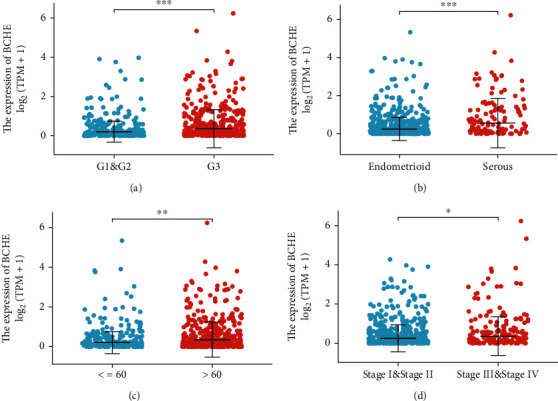
Differential BCHE expression in various clinicopathological parameters. Expression of BCHE was significantly different in (a) histological grade, (b) histological type, (c) age, and (d) clinical stage (^∗∗∗^*p* < 0.001; ^∗∗^*p* < 0.01; ^∗^*p* < 0.05).

Various clinical and pathological parameters of EC patients were compared with the expression levels of BCHE. Our results showed that BCHE was significantly different in histologic grade (*p* < 0.001, Figure 2(a)), histologic type (*p* < 0.001, Figure 2(b)), age (*p* = 0.002, Figure 2(c)), and clinical stage (*p* = 0.021, Figure 2(d)). Using logistic regression, we found that the BCHE expression level in EC was significantly correlated with histological type (serous vs. endometrioid, *p* value = 0.022), histological grade (G3 vs G1&G2, *p* value = 0.035), and age (>60 vs. ≤60, *p* value = 0.005) (Table 1).

### 3.3. Survival Outcome Analysis

**Figure 3:**
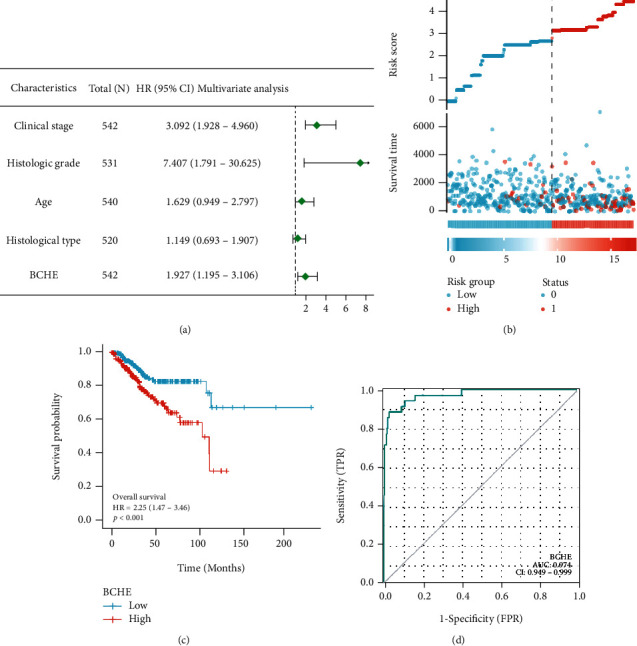
Survival analysis of BCHE expression. (a) Multivariate Cox analysis of BCHE expression and other clinicopathological variables. (b) BCHE expression distribution and survival status (0 = alive; 1 = death). (c) Levels of BCHE mRNA expression and overall survival. (d) ROC curves of BCHE.

**Figure 4:**
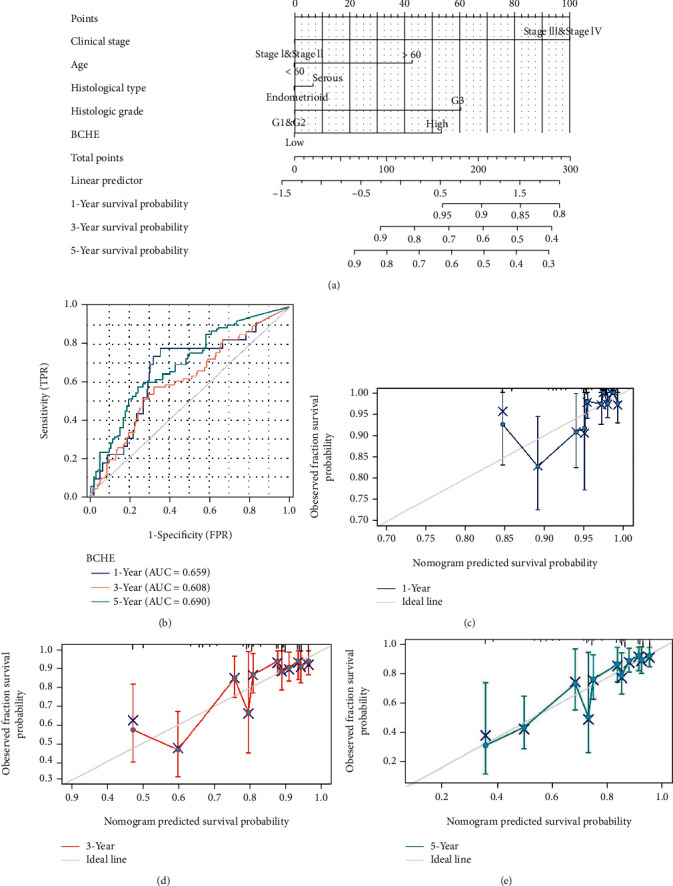
Nomogram construction and evaluation. (a) Nomogram construction based on BCHE and clinicopathological variables. (b) ROC curves of BCHE. (c) Calibration curves of 1 year. (d) Calibration curves of 3 years. (e) Calibration curves of 5 years.

As shown in Table 2, we explored the association between BCHE expression and overall survival in EC patients using the Cox analysis. The univariate Cox analysis revealed some factors including clinical stage (HR = 3.943, *p* < 0.001), histologic grade (HR = 11.401, *p* < 0.001), age (HR = 1.807, *p* = 0.013), histological type (HR = 2.874, *p* < 0.001), and BCHE expression (HR = 2.253, *p* < 0.001) significantly correlated with OS. Further, the multivariate Cox analysis, depicted as a forest boxplot in Figure 3(a), revealed that BCHE expression (*p* = 0.007) was an independent prognostic factor. The BCHE expression distribution, EC patients' survival status, and predicted risk scores based on the Cox models of BCHE are shown in Figure 3(b). Furthermore, based on K-M survival plots, the group with high BCHE expression had a lower overall survival rate (*p* < 0.001, Figure 3(c)). ROC curve showed that BCHE had promising prognostic power as its AUC was 0.974 (Figure 3(d)). Using this nomogram, we were able to calculate points and predict the survival rates for EC patients at 1, 3, and 5 years, improving the predictability (Figure 4(a)). The AUC values of this nomogram were 0.659, 0.608, and 0.690, respectively, and the results indicated a moderate accuracy of the prediction (Figure 4(b)). Our nomogram also performed well on 1-, 3-, and 5-year calibration curves (Figures 4(c)–4(e)).

### 3.4. Enrichment Analyses of BCHE

**Figure 5:**
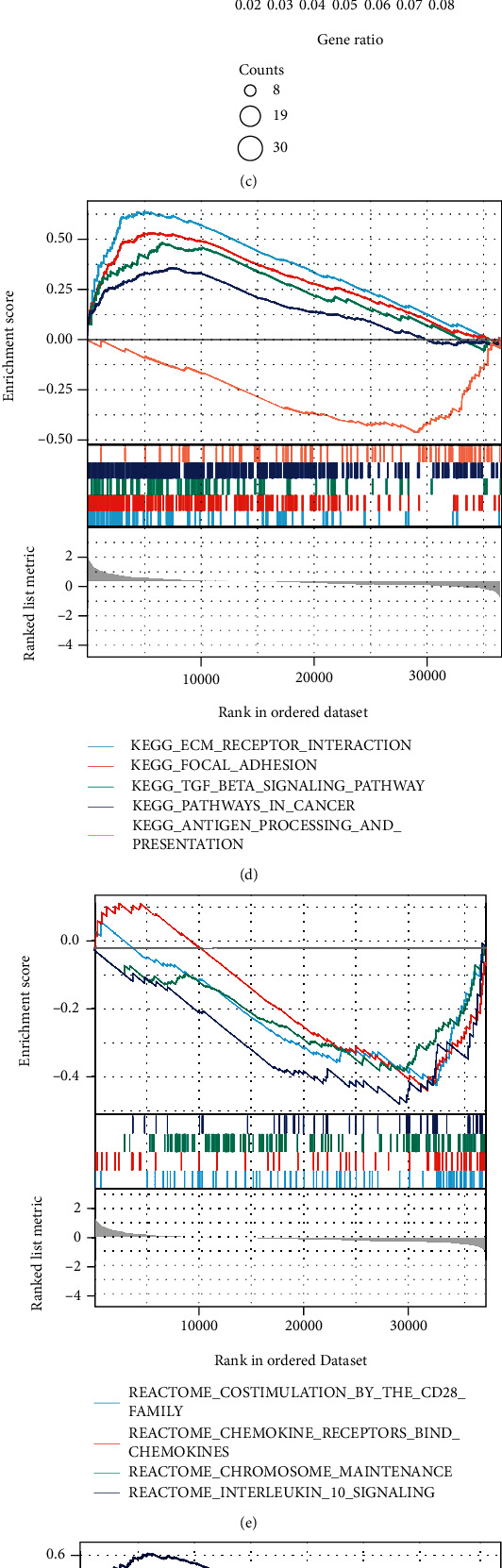
Function and pathway enrichment analyses of BCHE in EC. (a–c) Significant Gene Ontology terms associated with BCHE, including biological processes (BP), cell component (CC), and molecular function (MF). (d–g) Significant GSEA results associated with BCHE, including (d) KEGG pathways, (e) REACTOME pathways, (f) PID pathways, and (g) WP pathways.

The potential biological functions of BCHE were explored through GO and GSEA. As shown in Figures 5(a)–5(c), the BP, MF, and CC strongly associated with BCHE were transmembrane transport, signaling pathway, cell-cell adhesion, muscle system process, and heart process. As shown in Figures 5(d)–5(g), GSEA showed that the pathways regulating immune response, chromosome maintenance, cell adhesion, and interaction were critically important in EC patients.

### 3.5. Relationship of BCHE Expression with the Immune System and Tumor Microenvironment

**Figure 6:**
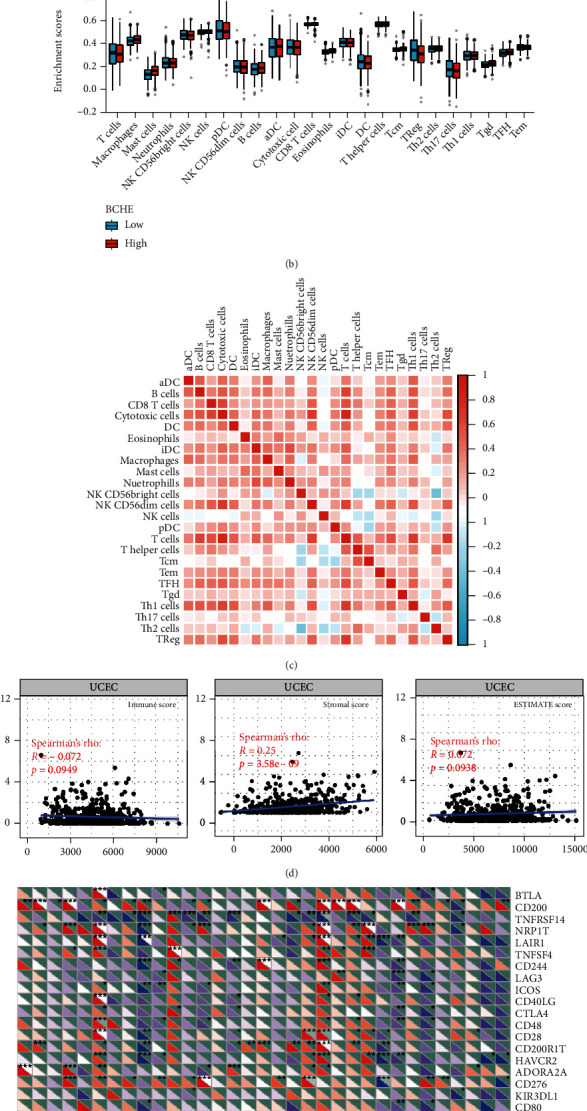
BCHE expression associated with the immune system and tumor microenvironment. (a) Correlations between BCHE expression and immune infiltration levels. (b) The varied proportions of 24 subtypes of immune cells in high and low BCHE expression groups in tumor samples. (c) Heatmap of 24 immune infiltration cells in tumor samples. (d) Relationships between BCHE and the immune microenvironment in EC. (e) Coexpression analysis of BCHE and immune checkpoint molecules in EC.

Independent tumor-infiltrating lymphocytes might play an important role in predicting overall survival [27]. As depicted in Figure 6(a), BCHE expression levels were positively correlated with mast cell counts, Tgd, eosinophils, Tcm, Th2 cells (all *p* = 0.002), TFH (*p* = 0.02), T helper cells (*p* < 0.001), CD8 T cells (*p* = 0.03), macrophages (*p* = 0.007), and NK cells (*p* = 0.009) and negatively correlated with the levels of Treg, Th17 cells (both *p* < 0.001), NK CD56 bright cells (*p* = 0.004), and NK CD56 dim cells (*p* = 0.02). Based on these results, BCHE might play a crucial role in immune infiltration of EC. In addition, we investigated whether tumor immune microenvironments differed between EC patients with different BCHE levels. As shown in Figure 6(b), compared to the low expression group, Tgd (*p* = 0.001), B cells (*p* = 0.039), eosinophils (*p* < 0.001), mast cells (*p* < 0.001), NK cells (*p* = 0.002), Tcm (*p* = 0.042), and TFH (*p* = 0.014) were increased in the high expression group, whereas Th17 (*p* = 0.025) and Treg (*p* value < 0.001) were decreased. Furthermore, we assessed possible correlations between 24 types of immune cells, and in Figure 6(c), we could see that the ratios of different tumor-infiltrating immune cell subpopulations exhibited weak to moderate correlations. Considering the microenvironment of EC, BCHE was markedly related to Stromal Score (*p* < 0.001); however, it was not linked to Immune Score (*p* = 0.0949) and Estimate Score (*p* = 0.0938, Figure 6(d)). According to a coexpression analysis between immune checkpoint molecules and BCHE, BCHE is highly correlated with CD28, ADORA2A, BTNL2, and TNFRSF18 in EC (all *p* < 0.05, Figure 6(e)).

### 3.6. Genetic Alteration Analysis

**Figure 7:**
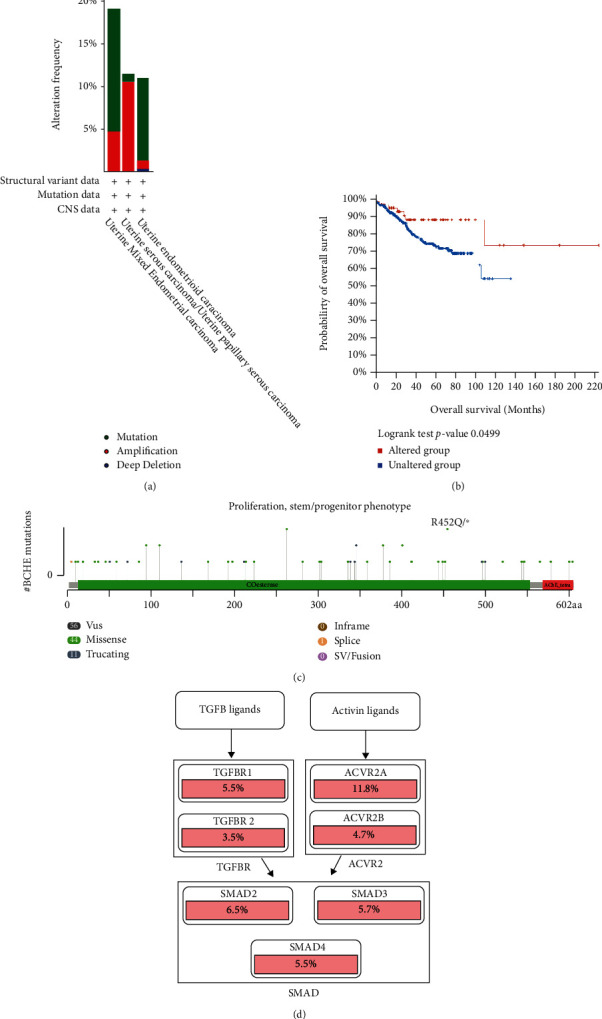
Mutation feature of BCHE in EC from TCGA cohort using the cBioPortal tool. (a) The alteration frequency with mutation type of BCHE in EC from TCGA cohorts. (b) K-M survival analysis of OS with or without BCHE alteration. (c) Mutation sites of BCHE in EC. (d) The relationship between the TGF-*β* signaling pathway and BCHE mutation.

Using the cBioPortal, we explored the mutational characters of BCHE in EC from TCGA and observed that the frequency of genetic alterations in BCHE was 11% (Figure 7(a)). As displayed in Figure 7(b), this indicated that EC cases with altered BCHE showed a better prognosis in OS (*p* = 0.049) than those without.  Figure 7(c) exhibits the mutation sites of BCHE in EC. The TGF-*β* signaling pathway was also associated with the BCHE mutation (Figure 7(d)). Therefore, genetic alterations of BCHE might play a big role in EC.

### 3.7. Relationships between BCHE and PPI, MSI, TMB, and TNC in EC

**Figure 8:**
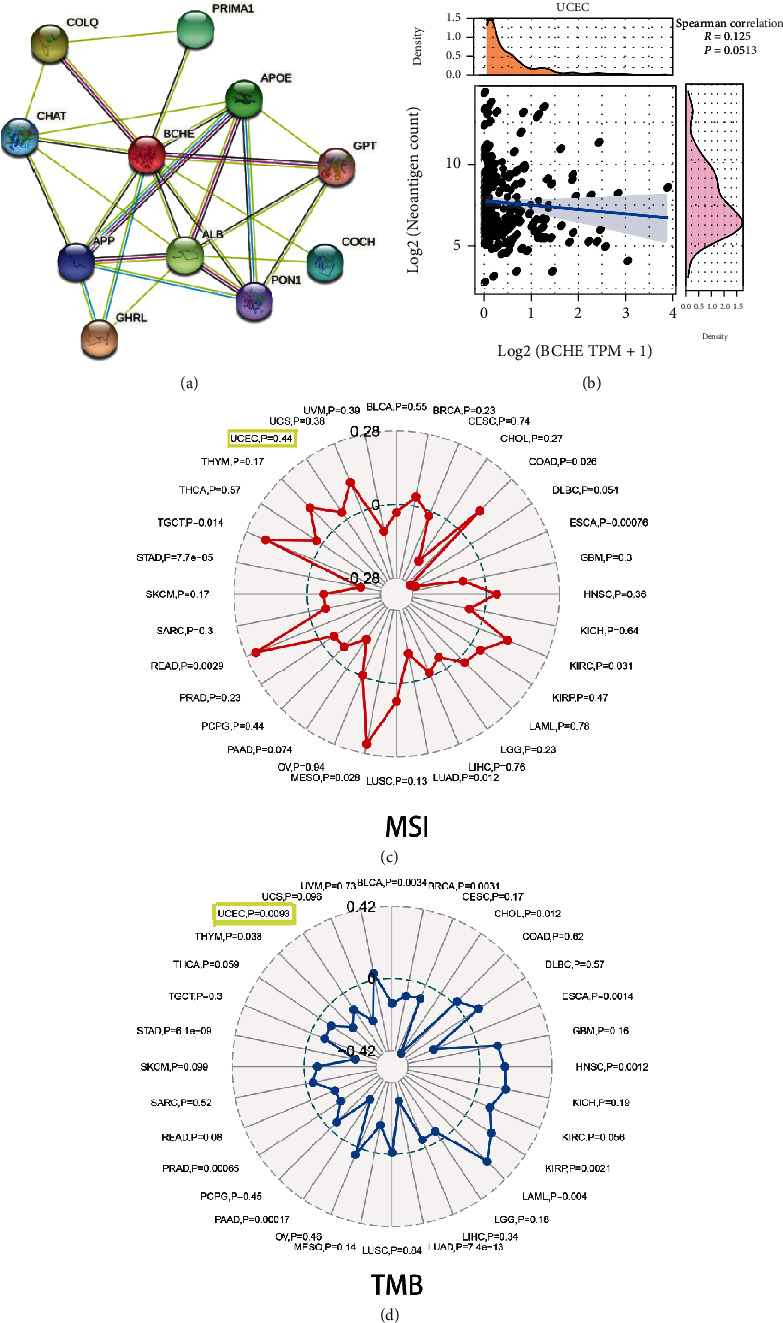
Relationships between BCHE and PPI, TNC, MSI, and TMB in EC. (a) PPI network. (b) Relationships between BCHE and TNC. (c) Relationships between BCHE and MSI. (d) Relationships between BCHE and TMB.

With the help of the online STRING database, BCHE was analyzed to find possible relationships with other genes in EC using PPI network analysis (Figure 8(a)). According to Figures 8(b)–8(d), our results revealed that BCHE expression was significantly correlated with TMB (*p* = 0.0093) in EC; however, it was not related to MSI (*p* = 0.44) and TNC (*p* = 0.0513).

## 4. Discussion

BCHE has been a very appealing biomarker in cancer diagnosis [27]; for instance, it has low expression in colorectal carcinoma [28] and high expression in ovarian cancer [29]. Moreover, in prostate cancer, BCHE expression was downregulated at early stages and upregulated at advanced stages [30]. Based on our results, the BCHE expression compared to normal tissues was high in 7 kinds of cancer and low in 20 kinds of cancer including endometrial carcinoma. Different levels of BCHE expression in different tumor types might reflect distinct functions and mechanisms.

In this article, we revealed the differential expression of BCHE in EC by using multiple publicly available databases. In contrast to normal tissues, endometrial tumors showed a low expression of BCHE and progressively higher expression as the disease progressed. BCHE expression was related to various tumor characteristics, and high BCHE expression was associated with a higher histological grade, type, and clinical stage than low BCHE expression. The GO and GSEA of this study suggested that upregulated BCHE was primarily related to transmembrane transport, signaling pathway, cell-cell adhesion, chromosome maintenance, and pathways regulating immune response which contained TGF-*β* signaling pathway and cancer immunotherapy by PD1 blockade. As a regulatory cytokine, TGF-*β* suppresses immune function in cancers and chronic viral infections [31–34]. In addition to altering the tumor microenvironment, TGF-*β* has an extensive immunosuppressive effect on natural killer (NK) cells, T cells, and myeloid cells [35]. TGF-*β*1 was found to be the key cytokine to modify antigen-driven PD-1 induction in a study [36]. In addition to inhibiting CD8+ T cells' ability to produce effector cytokines [37], TGF-*β*1 is known to suppress neighboring effector cells via both contact-independent and contact-dependent mechanisms to inhibit the development of CD4+ regulatory T cells (Tregs) [38, 39]. TGF-*β* signaling pathways playing a pivotal role in the regulation of cell proliferation and apoptosis of endometrial epithelial cells have been reported [40].

In addition, the potential connection between BCHE and immunity was explored, mostly in terms of immune infiltration, tumor microenvironment, and immune checkpoint molecules. Previous research has shown that the tumor microenvironment (TME) could facilitate tumor growth, metastasis, and resistance to chemotherapy and immunotherapy [41, 42]. In our study, BCHE was noticeably related to Stromal Score. As for immune infiltration, BCHE was significantly associated with Tgd, B cells, eosinophils, mast cells, NK cells, Tcm, TFH, Th17, and Treg. Studies have shown that Tregs accumulated in the tumor microenvironment and were increasingly recognized as a therapeutic target in cancer immunotherapy [43]. The TGF-*β* signaling pathway is essential for Treg differentiation and survival in the thymus and peripheral tissues. Thus, the regulation of TGF-*β* signaling pathways can be a noteworthy candidate for Treg control. Coexpression analysis of BCHE and immune checkpoint molecules presented that this gene was significantly related to CD28, ADORA2A, BTNL2, and TNFRSF18. According to all of these studies, BCHE was tightly associated with immunity in EC.

The nomogram has been widely used to assist clinical decision-making [44, 45]. Based on BCHE and four clinical parameters (age, clinical stage, histological grade, and type), we constructed a nomogram for predicting OS probabilities in EC. According to the AUCs and calibration curves for 1-, 3-, and 5-year periods, the prediction accuracy and performance of the nomogram were moderate.

The genetic alteration frequency of BCHE was 11% in TCGA-UCEC cohort, and the TGF-*β* signaling pathway was also associated with the BCHE mutation. As tumors develop, mutations accumulate and fuel the evolution of cancer. On the other hand, mutations can also hinder the evolution of tumors by triggering an immune response to the tumor if the mutations produce antitumor neoantigens, which are presented on the surface of the tumor cells and are recognized as “non-self” by immune cells [46]. In our results, the altered BCHE samples showed a better prognosis in OS compared with samples without alteration, indicating genetic alterations of BCHE might play an important role in EC. The MSI and TMB play an essential role in the growth and progression of cancer [47–49], and TMB has been the latest marker for evaluating the efficacy of PD-1 antibody immunotherapy. Our results found that BCHE was significantly correlated with TMB, but not with MSI.

## 5. Conclusions

To sum up, our outcomes revealed that BCHE might play an important role in the immune system and provide valuable insight into endometrial cancer prognoses. The TGF-*β* signaling pathway related to BCHE expression was worthy of attention. Moreover, there was a dramatic link between BCHE and TMB in endometrial carcinoma patients. In short, we expected our results to provide novel insights into EC immunotherapy for future research. In order to verify our results further, more clinical data and experiments are needed.

## Figures and Tables

**Table 1:** Association between BCHE expression and clinicopathological characteristics using logistic regression.

Characteristics	Total (*N*)	Odds ratio (OR)	*p* value
Clinical stage (stage III & stage IV vs. stage I & stage II)	543	1.408 (0.968-2.055)	0.075
Primary therapy outcome (PD vs. CR)	456	1.614 (0.655-4.193)	0.305
Race (Asian & Black or African American vs. White)	498	1.148 (0.766-1.723)	0.504
BMI (>30 vs. ≤30)	512	1.010 (0.710-1.438)	0.955
Histological type (serous vs. endometrioid)	521	1.639 (1.078-2.510)	**0.022**
Histologic grade (G3 vs. G1&G2)	532	1.454 (1.028-2.060)	**0.035**
Age (>60 vs. ≤60)	540	1.658 (1.169-2.358)	**0.005**
Diabetes (yes vs. no)	442	0.942 (0.619-1.432)	0.780

Bold font: *p* < 0.05.

**Table 2:** Correlation between overall survival and multivariable characteristics in TCGA patients via Cox regression analyses.

Characteristics	Total (*N*)	Univariate analysis		Multivariate analysis	
		Hazard ratio (95% CI)	*p* value	Hazard ratio (95% CI)	*p* value
Clinical stage	542				
Stage I & stage II	389	Reference			
Stage III & stage IV	153	3.943 (2.602-5.977)	**<0.001**	3.092 (1.928-4.960)	**<0.001**
Histologic grade	531				
G1	98	Reference			
G2&G3	433	11.401 (2.803-46.366)	**<0.001**	7.407 (1.791-30.625)	**0.006**
Surgical approach	520				
Minimally invasive	201	Reference			
Open	319	0.753 (0.489-1.160)	0.198		
Age	540				
≤60	206	Reference			
>60	334	1.807 (1.133-2.884)	**0.013**	1.629 (0.949-2.797)	0.077
Histological type	520				
Endometrioid	406	Reference			
Serous	114	2.874 (1.865-4.430)	**<0.001**	1.149 (0.693-1.907)	0.590
Menopause status	496				
Pre&peri	52	Reference			
Post	444	1.031 (0.497-2.139)	0.934		
BMI	511				
≤30	208	Reference			
>30	303	1.047 (0.682-1.606)	0.833		
BCHE	542				
Low	272	Reference			
High	270	2.253 (1.466-3.463)	**<0.001**	1.927 (1.195-3.106)	**0.007**

Bold font: *p* < 0.05.

## Data Availability

The data used to support the findings of this study are included within the article.

## Supplementary Materials

Supplementary 1 Supplementary Table 1: the full names of TCGA tumor abbreviation.

Supplementary 2 Figure S1: workflow chart.
