# Supplementary figures and images for: BCHE as a Prognostic Biomarker in Endometrial Cancer and Its Correlation with Immunity
Source: J Immunol Res. 2022 Jul 21;2022:6051092. doi: 10.1155/2022/6051092 (PMC9338740; doi:10.1155/2022/6051092)

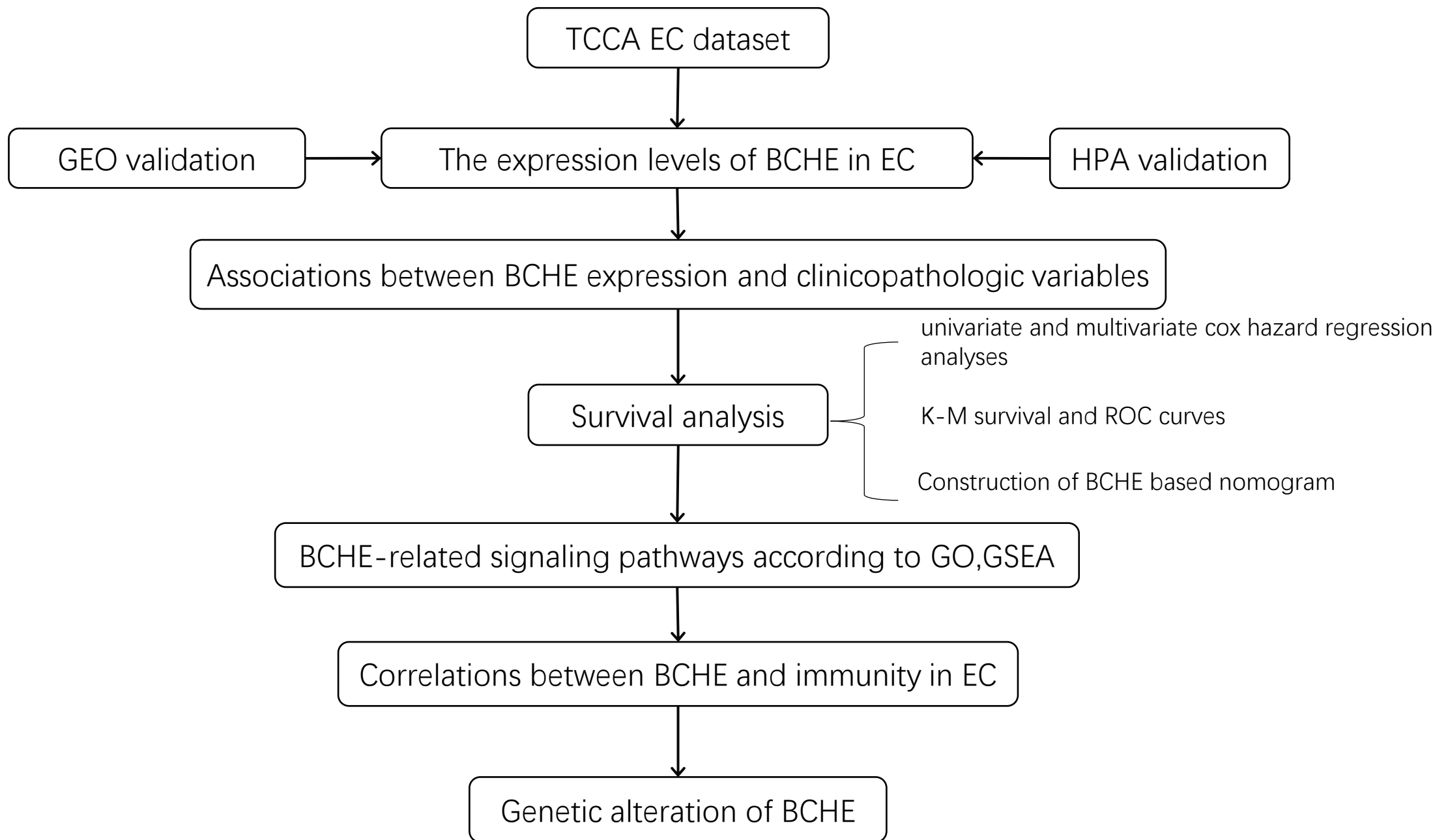

Supplement: Supplementary 2 — Figure S1: workflow chart. [file 6051092.f2.pdf]
